# Assessing workers with fibromyalgia: what should occupational physicians know?

**DOI:** 10.47626/1679-4435-2022-870

**Published:** 2023-08-08

**Authors:** Rafael Alves Cordeiro

**Affiliations:** 1 Serviço de Reumatologia, Hospital das Clínicas, Faculdade de Medicina, Universidade de São Paulo, São Paulo, Brazil

**Keywords:** fibromyalgia, chronic pain, central nervous system sensitization, occupational medicine, occupational health, fibromialgia, dor crônica, sensibilização do sistema nervoso central, medicina do trabalho, saúde do trabalhador

## Abstract

Fibromyalgia is a chronic pain syndrome with a complex multifactorial etiopathogenesis that more frequently affects women. Although widespread pain is the dominant feature, fibromyalgia incorporates a wide variety of symptoms, such as fatigue, unrefreshed sleep, and cognitive and mood disorders. Central sensitization to pain is a key element in the pathophysiology of this syndrome. Due to its prevalence and repercussions on quality of life and work productivity, fibromyalgia is a common condition in occupational medicine outpatient clinics. Thus, physicians must be attentive to its symptoms to facilitate diagnosis and management. This article will address basic topics about fibromyalgia, including: epidemiology, predisposing factors, pathophysiological considerations, clinical manifestations, classification criteria, differential diagnosis, basic principles of treatment, and the contribution of occupational physicians.

## Abstract (pt)

fibromialgia é uma síndrome dolorosa crônica, de etiopatogenia multifatorial complexa, que acomete com maior frequência mulheres. Embora a dor difusa seja o quadro dominante, a fibromialgia incorpora uma ampla variedade de sintomas, como fadiga, sono não reparador, queixas cognitivas e distúrbios de humor. A sensibilização central à dor é um elemento-chave no entendimento da fisiopatologia dessa síndrome. Por ser prevalente e ter reflexos na qualidade de vida e na produtividade no trabalho, a fibromialgia é uma condição recorrente em ambulatórios de saúde ocupacional. Nesse sentido, é importante que o médico do trabalho esteja atento aos sintomas da síndrome para que possa contribuir com seu diagnóstico e manejo. Neste artigo, serão abordados tópicos fundamentais para a compreensão da fibromialgia, incluindo epidemiologia, fatores predisponentes, considerações sobre a fisiopatologia, manifestações clínicas, critérios classificatórios, diagnósticos diferenciais, princípios básicos do tratamento e contribuições do médico do trabalho.

## INTRODUCTION

Fibromyalgia, a common chronic pain syndrome in clinical practice, is characterized by chronic widespread pain ≥ 3 months that is often associated with fatigue, unrefreshed sleep, cognitive complaints and mood changes.^1,2^ This disorder has a complex multifactorial etiopathogenesis. Current theory suggests that it is due to a pain processing dysfunction in the central nervous system, leading to amplified pain perception.^1,2^

The prevalence of symptoms compatible with fibromyalgia in the general population usually varies between 2 and 4% in most studies.^3^ However, the prevalence and proportionality between sexes vary according to classification criteria.^4^ In any case, it is clear that the condition is common, more often affects women and, not infrequently, appears in the occupational health setting due to presenteeism and absenteeism.

The challenge for occupational physicians is to suspect the syndrome, so that diagnosis can be confirmed when appropriate. Thus, appropriate treatment can be prescribed, thereby reducing the number of costly and unnecessary tests and procedures. In addition, occupational physicians can play an important role in fibromyalgia treatment through periodic occupational examinations or when workers seek out occupational health outpatient clinics. In such contexts, the physician should emphasize health education, regular exercise, sleep hygiene, weight loss (when relevant), adherence to pharmacological and non-pharmacological treatment, and remaining on the job market.

This article will address basic topics in fibromyalgia, including epidemiology, predisposing factors, pathophysiological considerations, clinical manifestations, classification criteria, differential diagnoses, basic principles of treatment and the contribution of occupational physicians.

## EPIDEMIOLOGY

Fibromyalgia is a common disorder whose worldwide distribution affects all races and socioeconomic groups. The literature suggests that the highest incidence is among women aged 30 to 50 years and that the prevalence increases with age.^5^ The prevalence of symptoms compatible with fibromyalgia in the general population usually varies between 2 and 4%.^3^ In Brazil, a study estimating the prevalence of rheumatic diseases in Montes Claros, state of Minas Gerais, using the Community Oriented Program for Control of Rheumatic Diseases questionnaire found a fibromyalgia prevalence of 2.5%.^6^

Although fibromyalgia predominantly affects women, the syndrome’s prevalence and the ratio between women and men vary according to the classification criteria. In 1990, the American College of Rheumatology (ACR) classification criteria for fibromyalgia were chronic widespread pain associated with pain on palpation in ≥ 11 of 18 tender points.^7^ New criteria were proposed in 2010 that do not involve counting tender points.^8,9,10^

## PREDISPOSING FACTORS

Factors that may predispose people to fibromyalgia include obesity and sedentary lifestyle, low socioeconomic status, sleep disturbances, a history of physical or sexual abuse, and diseases that cause peripheral pain, such as rheumatoid arthritis, systemic lupus erythematosus, and ankylosing spondylitis. However, fibromyalgia can occur in those with no apparent risk factors.^1^ Moreover, there are indications that genetic factors participate in fibromyalgia. Familial aggregation of cases has been demonstrated (ie, greater propensity for fibromyalgia in first-degree relatives of affected individuals) and polymorphisms of the catechol-O-methyltransferase enzyme gene and of genes related to neurotransmitter transporters/receptors have also been identified in patients with fibromyalgia.^12,13^

## PATHOPHYSIOLOGICAL CONSIDERATIONS

No single cause explains the appearance of fibromyalgia. Although the full details of its pathophysiology are still unknown, much progress has been made in recent decades toward understanding this syndrome.^14^ In general, the pain regulation/processing disorder that occurs in patients with fibromyalgia leads to central sensitization to pain, which is responsible for the amplification and perpetuation of pain perception.

Changes described in patients with fibromyalgia include: (1) hyperexcitability of nociceptive pathways (facilitation of painful afferents) with higher levels of substance P (a neuropeptide associated with chronic pain), neural growth factor, and glutamate; (2) dysfunction of descending pain inhibition pathways (endogenous analgesic response), including lower levels of antinociceptive neurotransmitters, such as noradrenaline, serotonin, dopamine, and gamma-aminobutyric acid; (3) greater activation of the somatosensory cortex, the anterior cingulate cortex, and the insula (areas related to pain perception), which has been shown in functional magnetic resonance imaging; and (4) changes in the hypothalamic-pituitary-adrenal axis and in the autonomic nervous system, which are related to sympathetic hyperactivity and increased plasma cortisol levels compared to healthy individuals.^14,15,16^

## CLINICAL MANIFESTATIONS

The main characteristic of fibromyalgia is widespread pain for ≥ 3 months, which is often accompanied by other complaints, such as fatigue, unrefreshed sleep, cognitive disorders (concentration, memory, and reasoning), and mood disorders (anxiety and depression).^1,2^ Patients with fibromyalgia may also have a variety of additional symptoms, such as paresthesia, sensation of edema in the extremities, dizziness, frequent headaches (tension and/or migraine), palpitations, subjective weakness, chronic pelvic pain, bladder symptoms, dyspepsia, irritable bowel syndrome, temporomandibular joint dysfunction, etc.^1,2,8^

## DIAGNOSIS AND CLASSIFICATION CRITERIA

Fibromyalgia diagnosis is primarily clinical, with widespread pain playing a key role for the physician to suspect the syndrome. Sleep disturbances, cognition complaints, and fatigue should be considered not only in diagnosis, but in severity assessment.^17^ Psychological variables, such as depression and anxiety, are associated with lower functional capacity and greater perceived severity; hence, these factors should be actively investigated.^18^ Given the lack of laboratory markers or imaging findings, fibromyalgia is diagnosed according to clinical judgment after considering the differential diagnoses in the evaluation.^17^

Classification criteria are designed for clinical and epidemiological studies to ensure uniformity among the included patients. Such criteria are often extrapolated to clinical practice, although they are not specifically intended for patient diagnosis, which should be the physician’s responsibility.^19^

**Figure 1:**
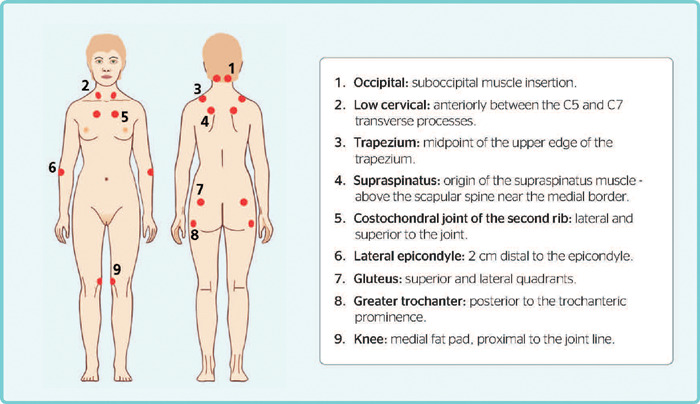
Location of tender points according to American College of Rheumatology classification criteria, 1990.

In 1990, the ACR published an initial set of criteria for diagnosing fibromyalgia (Chart 1).^7^ According to these criteria, the patient would have to present chronic widespread pain (defined as pain above and below the waist, on the right and left sides of the body, and in the axial skeleton for ≥ 3 months) in association with pain on palpation at least 11 of 18 tender points (Figure 1).^7^ Despite having helped standardize patients for inclusion in fibromyalgia studies, these criteria have been criticized due to their emphasis on widespread pain, without considering fatigue, sleep disturbances, or other frequent somatic symptoms in the syndrome. The mandatory evaluation of tender points was also criticized, since many health professionals did not have adequate training or experience to recognize them.^17^

**Chart 1:** American College of Rheumatology 1990 classification criteria for fibromyalgia^7^

For classification purposes, patients will have fibromyalgia if both criteria are met:
1. History of widespread pain (≥ 3 months)
2. Pain on palpation of at least 11 of the 18 tender points Digital palpation should involve a force of approximately 4 kg.
A second clinical disorder does not exclude the diagnosis of fibromyalgia.

In 2010, the ACR developed new criteria,^8^ which were modified in 2011.^9^ These criteria replaced tender point assessment with a widespread pain index and included a symptom severity score, emphasizing other frequent complaints in fibromyalgia in addition to widespread pain (Charts 2 and 3). In 2016, the 2010/2011 criteria were further revised, adding slight changes^10^ (Chart 4).

**Chart 2:** Widespread pain index^9^

Indicate the areas where you felt pain over the last week:					
Area	Yes	No	Area	Yes	No
Jaw, right			Jaw, left		
Shoulder girdle, right			Shoulder girdle, left		
Upper arm, right			Upper arm, left		
Lower arm, right			Lower arm, left		
Hip, right			Hip, left		
Upper leg, right			Upper leg, left		
Lower leg, right			Lower leg, left		
Neck			Upper back		
Chest			Lower back		
Abdomen					

The result is the sum of the indicated items (0 to 19).

**Chart 3:** Symptom severity score^9^

Indicate the number corresponding to the intensity of the symptoms you have felt in the last week:				
Fatigue (tiredness when performing activities)	None 0	Mild 1	Moderate 2	Severe 3
Unrefreshed sleep (waking up tired)	None 0	Mild 1	Moderate 2	Severe 3
Cognitive symptoms (memory and concentration difficulties)	None 0	Mild 1	Moderate 2	Severe 3

Indicate the number corresponding to the number of symptoms you have experienced in the last 6 months:				
Headache Pain or cramps in the lower abdomen, Depression symptoms	None 0	1 of these symptoms 1	2 of these symptoms 2	3 of these symptoms 3

The result is the sum of the indicated levels (0 to 12).

**Chart 4:** Revised American College of Rheumatology Criteria, 2016^10^

The patient meets the modified fibromyalgia criteria (2016) if the first 3 conditions are met:
1. Widespread pain, defined as pain in at least 4 of 5 regions (axial, upper right, upper left, lower right and lower left).
2. Symptoms must have been present at a similar level for ≥ 3 months.
3. WPI ≥ 7 and SSS ≥ 5 or WPI 4-6 and SSS ≥ 9.
4. A diagnosis of fibromyalgia is valid regardless of other diagnoses. A diagnosis of fibromyalgia does not preclude other clinically significant diseases.

WPI = widespread pain index; SSS = symptom severity score.

For clinical practice, rather than memorizing the different classification criteria, occupational physicians should be aware of pain characteristics (diffuse and chronic); associated symptoms (fatigue, disturbances in sleep, cognition, mood, etc.); and possible differential or coexisting diagnoses (detailed clinical evaluation).

## DIFFERENTIAL DIAGNOSIS

Most differential diagnoses can be ruled out through clinical history and a detailed physical examination. Generally, numerous laboratory and/or imaging tests are not required. Another issue to be considered is that fibromyalgia can coincide with several other diagnoses, including conditions that cause chronic pain. Thus, it is a good idea for occupational physicians to have a rheumatologist colleague they can confer with regarding differential or concomitant diagnoses.

The following differential diagnoses should be mentioned: (1) autoimmune rheumatic diseases (rheumatoid arthritis, systemic lupus erythematosus, Sjögren syndrome, spondyloarthritis, and polymyalgia rheumatica); (2) myopathies (inflammatory, metabolic, or statin); (3) osteoarthritis, myofascial syndrome, and soft tissue rheumatism, such as bursitis and tendinitis; (4) endocrine disorders, such as hypothyroidism, hyperparathyroidism, and Cushing’s syndrome; (5) infectious diseases, such as chronic viral hepatitis and Chikungunya (chronic phase); and (6) peripheral neuropathies.^20,21^

In the initial laboratory evaluation of patients with suspected fibromyalgia, some authors suggest requesting a complete blood count and tests for inflammatory activity (C-reactive protein and erythrocyte sedimentation rate), thyroid stimulating hormone, serum calcium, and creatine phosphokinase.^20^ These results should be normal in patients with fibromyalgia and no concomitant conditions.

Other tests may be requested for additional suspicions raised in anamnesis and clinical evaluation. It is worth mentioning that laboratory tests for autoimmune diseases, such as rheumatoid factor and antinuclear antibodies, should not be routinely requested, since they can be positive even in healthy individuals. These tests could be considered in the appropriate clinical context, eg, when findings such as synovitis and skin lesions suggest autoimmune disease.^22^

Common diseases in the general population, such as osteoarthritis, tendinitis, bursitis, and myofascial syndrome, are also found in patients with fibromyalgia. Thus, any peripheral pain generators should also be treated in patients with fibromyalgia.^20,21^

## BASIC TREATMENT PRINCIPLES

Fibromyalgia treatment should follow a multimodal strategy that includes both non-pharmacological and pharmacological strategies.^1^ The main goals of fibromyalgia treatment are (1) to minimize pain and alleviate associated symptoms; (2) to improve quality of life; (3) to promote healthy living habits; and (4) to keep the patient productive and in the labor market.

According to the European Alliance of Associations for Rheumatology’s (EULAR) revised fibromyalgia recommendations, optimal treatment requires early diagnosis. A complete understanding of fibromyalgia involves a comprehensive assessment of pain, function, and psychosocial context.^23^ Initial treatment should always include non-pharmacological strategies. If the response is insufficient, treatment should combine non-pharmacological and pharmacological strategies, which should be individualized according to pain intensity and associated characteristics (depression, fatigue, sleep disturbances, and comorbidities).^23^

## NON-PHARMACOLOGICAL STRATEGIES

Patient education should include the following: (1) information about diagnosis and treatment of the syndrome, (2) emphasis on the benign and non-deforming nature of the condition, (3) the importance of exercising and staying active to control symptoms, (4) the importance of sleep hygiene, (5) the importance of weight loss (when relevant), and (6) guidance about active patient participation in treatment, as well as adherence to pharmacological and non-pharmacological strategies.^1,23^

Based on meta-analyses, the EULAR guidelines strongly recommend regular exercise (aerobics, stretching, and muscle strengthening), mainly due to their effect on pain, functionality, and well-being, as well as their availability and relatively low cost.^23^ Although it is common for patients to experience some degree of worsening pain and fatigue once they begin a physical activity program, they should be encouraged to persist and gradually increase exercise intensity, since physical activity is fundamental to symptom reduction.

Cognitive behavioral therapy is particularly helpful for those with mood disorders. It helps develop coping and self-efficacy strategies and also helps reduce negative beliefs, catastrophizing, and hypervigilance.^23^ Acupuncture, hydrotherapy, meditative movement therapies (qigong, yoga, tai chi), and mindfulness-based stress reduction may also be helpful.^23^

## PHARMACOLOGICAL STRATEGIES (CHART 5)

**Chart 5:** Medications used to treat fibromyalgia^23^*

→ Amitriptyline (tricyclic antidepressant)
→ Duloxetine and milnacipran (dual serotonin and norepinephrine reuptake inhibitors)
→ Pregabalin and gabapentin (alpha-2 binding gabapentinoid anticonvulsants)
→ Tramadol (weak opioid). In fibromyalgia, it has been postulated that tramadol’s analgesia mechanism is a mild serotonin and noradrenaline reuptake inhibition effect, rather than opioid receptor agonism.
→ Cyclobenzaprine (centrally acting muscle relaxant in the tricyclic group; has no effect on depression)

* Details on dosage, dose increments, and adverse effects of these medications are beyond the scope of this article.

Medications should be prescribed according to the predominant symptoms, potential adverse effects (considering comorbidities), and costs.^1,23^ In general, medications are administered in low doses. The doses are gradually increased according to clinical response and patient tolerance. Care should be taken with amitriptyline, tramadol, pregabalin, gabapentin, and cyclobenzaprine for individuals who drive to work or who work at heights or in jobs with a high risk of accidents, since these drugs can cause drowsiness (administration schedules must be rigorously adjusted according to the half-life of each medication).

Simple analgesics and non-hormonal antiinflammatory agents are not effective for central sensitization, but they can be useful in specific situations involving peripheral pain generators, such as tendinitis and bursitis.^1,23^ The EULAR guidelines strongly discourage the use of growth hormones, sodium oxybate, strong opioids, and glucocorticoids due to their lack of efficacy in fibromyalgia and the high risk of side effects.^23^

## THE CONTRIBUTION OF OCCUPATIONAL PHYSICIANS

The inability to work due to pain can be influenced by a complex interaction between several factors: past experiences, education, socioeconomic status, job satisfaction, psychological distress, fatigue, personal values, cultural context, and availability of financial compensation.^24^

Patients with fibromyalgia report that symptoms adversely affect their quality of life and ability to work.^25^ However, determining disability is particularly difficult, since pain is a subjective sensation that, in fibromyalgia, cannot be understood within a classic model of disease and tissue damage. Self-perceived disability sometimes seems to be the main difference between those who stay at work and those who seek insurance benefits.^24^

Thus, when employees with fibromyalgia report having impaired work capacity, occupational physicians must assess: (1) whether the employee is undergoing adequate therapeutic follow-up; (2) whether the employee is adhering to pharmacological and non-pharmacological treatment; (3) whether there are significant stressors at work that could exacerbate fibromyalgia symptoms; (4) whether there is an obvious mismatch between work demands and the employee’s age; and (5) whether the employee has been experiencing severe psychosocial stressors or could have co-occurring psychiatric diagnoses. All of these issues are important to adequately guide interventions. They are also essential for making recommendations, adaptations, and restrictions to the work routine, as well as the decision to keep employees at work or send them on temporary sick leave.

Another opportunity for occupational physicians is upon diagnosis (or at least suspicion) of fibromyalgia in workers who are unaware of the nature of their symptoms. It is not uncommon for patients to take years to get a diagnosis and to seek different specialists for each symptom they present. This not only delays more effective treatment, but can also entail numerous costly and unnecessary tests.^26^ Some studies have suggested that establishing a diagnosis of fibromyalgia leads to lower resource use and lower overall health care costs.^27,28^ In the context of occupational medicine, this would help the sustainability of corporate health insurance plans.

Periodic health examinations are component of occupational medicine and have a prominent role in the Brazilian Program for Medical Control of Occupational Health (*PCMSO*). Through periodic clinical evaluations, occupational physicians can identify health changes, whether related or not to work activity. In addition to suspecting fibromyalgia syndrome in employees complaining of widespread chronic pain, occupational physicians can, during periodic examinations, reiterate important aspects of patient education among those who have been diagnosed. In these cases, emphasis should be placed on sleep hygiene, treatment adherence, and regular physical activity. It is worth mentioning that some studies recommend exercise not only for fibromyalgia symptom control, but as a long-term strategy for coping with work.^29,30^

## CONCLUSIONS

The diagnosis and treatment of fibromyalgia are a challenge for both patients and health professionals. Efforts should be directed towards recognizing the syndrome to minimize pain and alleviate associated symptoms, improve quality of life, promote healthy habits, and keep patients productive and in the job market. Occupational physicians can play an important role in this regard.
